# Modelling the impact of hailstones on flat steel roofing membranes for residential buildings

**DOI:** 10.1038/s41598-022-24375-3

**Published:** 2022-11-18

**Authors:** Mehmet E. Uz

**Affiliations:** Civil Engineering, Faculty of Engineering, Adnan Menderes University, Aydın, Turkey

**Keywords:** Civil engineering, Structural materials

## Abstract

Metal roof panels are commonly used on residential and commercial buildings. Steel panels exposed to hail have not yet been adequately tested for dent resistance. A finite element model (FEM) was used to analyze the entire test setup. To compare artificial hailstones with natural hailstones, which remained intact after impact, different steel sheets were struck by different sizes of artificial hailstones at different terminal velocities. The simulation and the material properties are assessed by comparing the experimental results with the FE model. An equation to predict the dent depth based on kinetic energy and stress is also presented. The results of this study provide a better understanding of the failure modes of hail and roof panels and their effects on dent resistance. In this study, the results of observations and numerical simulations agreed well with those of analytical models. The result is that the proposed equation overestimates the dent depths compared to the dent depths obtained with finite element models, while the equation leads to an underestimation of the dent depths found in the steel sheets.

## Introduction

Hailstorms can severely damage roofs. Depending on the air flow, hailstones up to 45 mm in diameter can be observed during hailstorms. Hailstorm-related damage often involves roof damage. The literature still suggests that hailstorms can cause significant damage to roofs. If there is no leakage or other visible damage, hail damage is not visible on a roof. In addition, hail that remains solid after impact causes significant damage. Simulation of the behavior of hailstones through their impact is crucial to accurately evaluate hail destruction on roof materials. Since natural hailstones do not break, they cause more damage because no energy is lost by breaking up^1^. Furthermore, a method needs to be developed that allows artificial hailstones to stay whole during a high-speed impact. Liquid nitrogen is used to create hailstones that can remain whole after high-velocity impact. Similar to natural hailstones, artificial hailstones are uniform and dense. For an accurate determination of the dent resistance of steel plates, especially under dynamic conditions, it is necessary to obtain the realistic behavior of hail during impact. The risk of failure of a roof system must be evaluated together with significant factors such as hail size, hail speed, roof plate form and ultimate stress. Until now, there have been no studies of this kind. In this study, computation and experimental testing are employed to determine dent resistance. FE simulations were carried out via Abaqus software^2^. A novel aspect of this study is to perform a finite element analysis of the hailstorm, validated by laboratory tests. The main focus is on the depth of the dents in the steel sheets after the hailstorm, as they vary in thickness with respect to the direction of loading.

### Using hail

When artificial hailstones freeze from the outside in, the air gathers in the outer shell. As a result of trapped air that is retained locally, hailstones have a weak point. Kim et al.^3^ and Flüeler et al.^4^ used shallow layering to produce synthetic hail, which was replicated by Tippmann^5^. Real hailstones have a layered structure from base to tip that resembles layers of an onion. Therefore, all hailstones produced with this method break when hitting a hard surface. The immersion method of Allaby and Garratt^6^ involves growing embryos on dry ice, enclosing them in a ping-pong ball with a hole at the top and freezing them in boiling water. In this process, the hailstones were spherical, and their outer layer was transparent. One of the twelve models tested survived the first impact test, although none of them were perfectly round. After impact, an ice sphere can maintain its solidity by increasing its tensile strength. A study conducted by Gold^7^ shows that the material known as pykrete has very different properties from ice. To increase the tensile strength of the ice, cotton fibers or polypropylene fibers were included. Artificial hailstones were strengthened with PVA-based adhesive and microfibers, but their parameters were not considerably altered, according to Wu^8^. Artificial hailstones have been launched at velocities of nearly 30 m/s onto G300 steel plates with a thickness of 0.55 mm. Only an ice ball made of 88% boiling water and PVA glue passed the impact test. The admixture of PVA in hail causes it to behave like rubber^9,10^. Here, for the first time reported in the literature, artificial hailstones were made with liquid nitrogen and water. A mixture of 88% demineralized water and 12% PVA was used to create the artificial hailstones. When impacting steel roofs with higher terminal velocities, artificial ice hailstones created from liquid nitrogen remained intact.

### Roof panels

The behavior of roof panels in hail has not yet been studied in detail. Koontz^11^ examined factors such as the size of the hail, the angle of impact, the aging of the panels, the type of roof, the weather and the surface to describe how hail damages roofs. In a study of hailstone damage thresholds on metal plates and impact tests on aging roof materials, Timothy et al.^12^ investigated the destruction levels. The central plastic deformation of the plate was predicted using an accurate model developed by Mohotti et al.^13^ In their study, the time history of the deformation is foreseen methodically, arithmetically, and empirically. However, the projectiles were made of a cylindrical steel rod with a diameter of 37 mm and a flat impact plane. They concluded that all the kinetic energy applied to the sheet during impact was entirely converted into permanent deformation. According to Calder and Goldsmith^14^, energy losses are only a small factor in deformation caused by impacts. Depending on the shape of the projectile and the softness of the object, some researchers have also studied predicted force functions^15–18^.

## Methods of creating artificial hailstones

**Figure 1:**
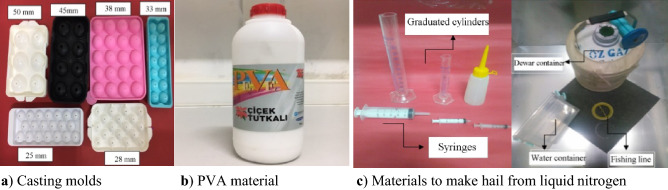
Photos of materials to make hailstones of different sizes^9,20^.

Usually, placeholder hailstones of different shapes, sizes and masses are thrown or dropped at different velocities. The fall velocity of natural hail was most recently estimated by Heymsfield et al.^19^ The hailstones were formed with 12% polyvinyl acetate (PVA) and nitrogen. A mixture of liquid nitrogen and demineralized water was used to produce artificial hailstones that were solid, in what is considered to be a new method. The following is an explanation of the equipment and processes used to produce artificial hailstones. A tank was required to transport liquid nitrogen without harming the surroundings. This method of containing liquid nitrogen protects it from evaporation and limits dangerous conditions. Since the liquid nitrogen is transferred from the tank to the Dewar container, it can be used conveniently every time an experiment is carried out. Pure water was stored in tanks to prevent decomposition. To maintain consistent dipping, the liquid nitrogen is immersed in water tanks in the Dewar vessel. The embryo was exposed to demineralized water droplets or sand particles. The embryo was quickly immersed in liquid nitrogen in a Dewar vessel. The water in the container then suddenly froze. Artificial hailstones made with PVA have sizes of 37.5, 45 and 50.8 mm (see Fig. 1a). The tools required to produce these artificial hailstones are shown in Fig. 1b and c. The steel panels were tested six times with artificial hailstones of different sizes. In this study, 226 impact tests were conducted. The validity of the equation obtained in this study was assessed using hailstones made of PVA and liquid nitrogen.

The projectile integrity in this study was classified into four cases: intact, partly intact, major fragmentation and shattered. In this study, the impact velocity of hailstones was measured by sensors and cameras at different sheet thicknesses. For hailstones with different diameters and densities, the final velocity can be determined by adjusting the pressure in the gas tank. Ten different plates were used in this study. As reported by the supplier, all plates have a yield strength of 300 MPa and measure 1 m × 1 m in size.

In this study, ten sheets were used with thicknesses of 0.3, 0.45, 0.6, 0.7, 0.8, and 1.0 mm. Each test was conducted at perpendicular angles. In this study, only the depths of dents induced by nitrogen ice balls and PVA that did not break were examined.

### Dynamic testing equipment

**Figure 2:**
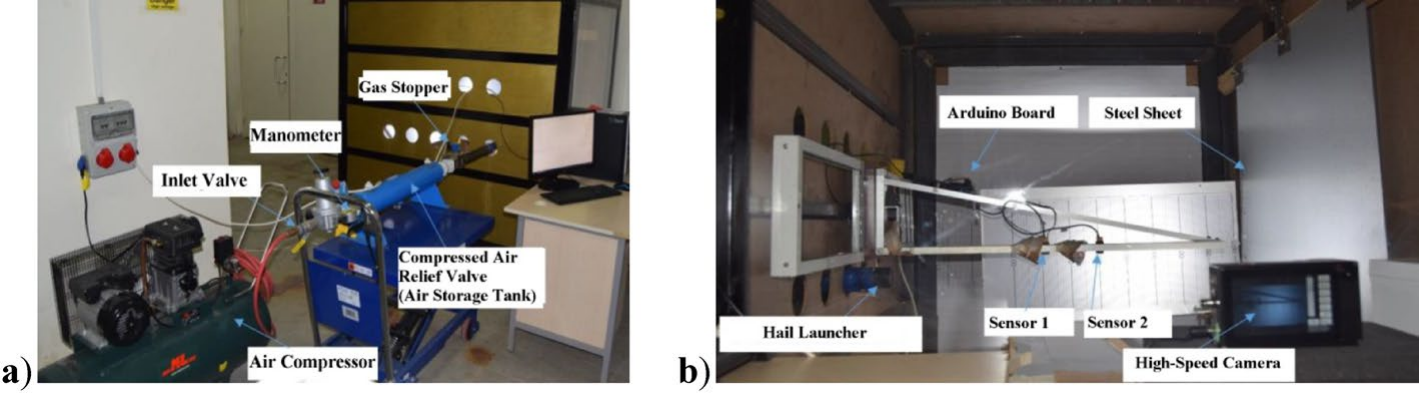
Setup using (**a**) a hail cannon and (**b**) a camera and velocity probes.

The main components of the test setup are a hail cannon, a protective housing, and a measuring device. Figure 2 shows the setup. Prior to loading the artificial hailstones into the hail cannon, it is necessary to determine the mass, volume, and density of the hailstones. The hail cannon is shown in Fig. 2a.

**Figure 3:**
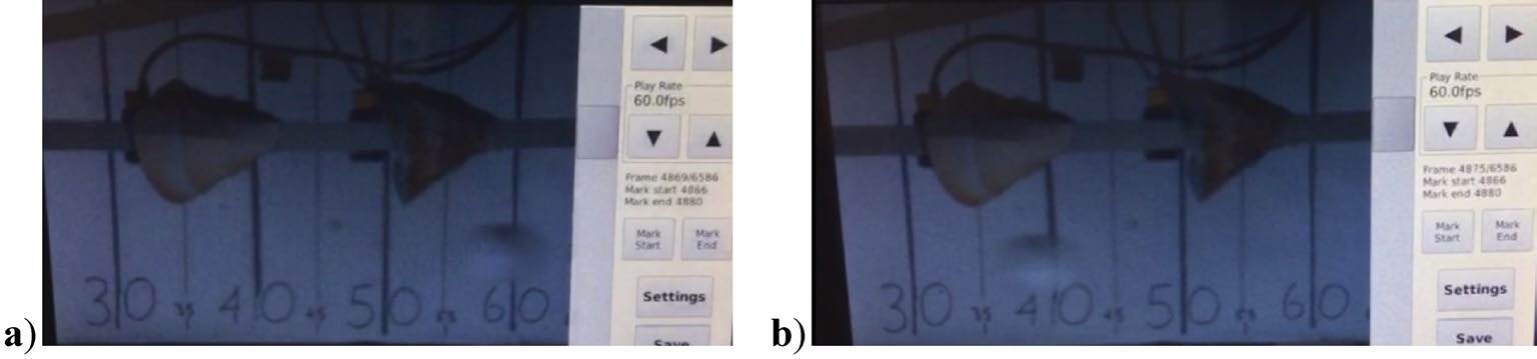
Images of hailstones made of PVA with a diameter of 38 mm (**a**) before impact and (**b**) after impact.

Observation and recording of the impact are possible due to the large glass window in the protection unit. There are five holes in two rows in the protection unit, which can be used to aim the hail cannon at different areas of the steel sheet. The speed sensors are mounted on rails attached to the protection unit. A high-speed camera was used to detect and measure the speed of hailstones (see Fig. 2b). To make efficient use of a high-speed camera, a 185-W lamp LED was used, powered by DC rather than AC, which reduced the flicker in the videos taken at high frames per second. To accurately measure the trajectory of the artificial hailstones, a steel frame ruler was placed along the trajectory from the barrel. Using trial and error, a frame rate of 1000 frames per second was used based on the quality of the image and the time it took to capture it. The impact velocity of an artificial hailstone can be determined using the scale as shown in Fig. 3.

In addition, high-speed cameras can be used to observe and assess hailstones upon impact. The camera's speed measurements were confirmed using MATLAB. A rail system was equipped with two laser sensors placed at a specific distance from one another. The projectile is detected by each sensor in turn. A time duration is displayed when the Arduino board detects the first and second sensors. The test board is replaced after ten shots, and the depth and diameter of the dent are measured. To define the diameter of the dent, the distance between the edges of each axis was evaluated with a Vernier caliper. Currently, the configuration and angle of steel sheets are not considered, as this is outside the scope of the study.

### Dent depth equation

The impact energy of an intact artificial hailstone is mainly converted to the plastic deformation energy of the dented sheet, the rebounded energy, and the flexural vibration energy as other forms of energy loss such as heat and sound are negligible. According to Patil and Higgs^22^,the collision damage equation can be written as Eq. (1).

1 $${E}_{Impact}={E}_{Vibration}+{E}_{Plastic}+{E}_{Rebound}$$

As ${E}_{Rebound}$ converges to zero $\left({E}_{Rebound}\cong 0\right)$, it is considered an insignificant variable, while the vibration energy ${E}_{vibration}$ represents the largest proportion of the impact energy. During impact, the plastic energy $\left({E}_{Plastic}\right)$ of a material is related to its yield stress and volume change. As the thickness of the material remains constant during impact, the plastic energy can be provided by Eq. (2):

2 $${E}_{Plastic}={\sigma }_{y}t\Delta A$$

It is possible to calculate the area changed after impact according to Eq. (3). A simplified depressed area is signified by the radius r before impact, and the radius of the hailstone is represented by $R$. The indentation depth is denoted by $D$. The initial (original) depressed area is given as follows.

3 $$\Delta A={{A}_{f}-A}_{0}$$

4 $${A}_{0}=\pi {r}^{2}=\pi \left({R}^{2}-{\left(R-D\right)}^{2}\right)=\pi \left(2RD-{D}^{2}\right)$$

Equation (5) gives the distorted area after the impact. Equations (6) and (7) are used to calculate the modified area and plastic energy, respectively.

5 $${A}_{f}={\int }_{\theta }^{\frac{\pi }{2}}2\pi rR{d}_{\theta }=2\pi RD$$

6 $$\Delta A=\pi {D}^{2}$$

7 $${E}_{Plastic}=\pi {D}^{2}{\sigma }_{y}t$$

It is assumed that the external vibration and the external frequency $\left(w\right)$ of the hailstone produced by the impact are zero since the impact is almost instantaneous. In this case, due to the stability of the system, the pressure gap can be expressed by Eq. (8) for a sinusoidal steady-state response.

8 $$x={u}_{st}\left|{H}_{jw}\right|\mathrm{cos}(\omega t+\Phi )$$

When $$\omega =0$$,

$$\left|{H}_{jw}\right|=\sqrt{\frac{1}{{\left(1-\left(\frac{\omega }{{\omega }_{n}}\right)\right)}^{2}+{\left(2\delta \left(\frac{\omega }{{\omega }_{n}}\right)\right)}^{2}}}=1$$

$$\mathrm{tan}\left(\Phi \right)=\frac{2\delta \left(\frac{\omega }{{\omega }_{n}}\right)}{1-{\left(\frac{\omega }{{\omega }_{n}}\right)}^{2}}=0 \to {\Phi }^{o}={0}^{o}$$

9 $$\left|{H}_{jw}\right|\mathrm{cos}(\omega t+\Phi )\cong 1$$

where $\left|{H}_{jw}\right|$ in Eq. (9) represents the dynamic amplification factor. This means that the vibration energy equals:

10 $${E}_{Vibration}=\frac{k}{2}.\frac{{F}^{2}}{{k}^{2}}=\frac{{F}^{2}}{2k}$$

Flat steel sheets have a flexural stiffness $\left(k\right)$ proportional to their thickness $\left(t\right)$ and the length $\left(h\right)$ cubed but inversely proportional to the spacing between their battens cubed.

11 $$k=\frac{{Eth}^{3}}{{l}^{3}}$$

The symbol $\varphi$ symbolizes the diameter of a hailstone (2R). To explain the vibration energy, Eq. (12) can be substituted into Eq. (10).

12 $$F=\sqrt{\frac{{{\sigma }_{y}}^{2}{\pi }^{2}{\varphi }^{4}}{16}}$$

13 $${E}_{Vibration}=\frac{{{\sigma }_{y}}^{2}{\pi }^{2}{\varphi }^{4}{l}^{3}}{{32Eth}^{3}}$$

The final step is to rewrite Eq. (1) to obtain dent depth:

14 $$D=\sqrt{\frac{m{V}^{2}}{2t\pi {\sigma }_{y}}-\frac{{\sigma }_{y}\pi {\varphi }^{4}{l}^{3}}{32E{t}^{2}{h}^{3}}}$$

To optimize the dent depth in Eq. (14), the rebounded energy and the compressive area can be considered by using the following equation:

15 $$D=\sqrt{\alpha \times \frac{m{V}^{2}}{2t\pi {\sigma }_{y}}-\beta \times \frac{{\sigma }_{y}\pi {\varphi }^{4}{l}^{3}}{32E{t}^{2}{h}^{3}}}$$

where $\alpha$ and β denote the rebound energy and the pressure area, respectively. The coefficient of restitution (COR) is usually referred to as the cause of energy dissipation during an impact. The COR is calculated as follows:

16 $$COR=\frac{Rebound\,\, Velocity}{Impact \,\,Velocity}$$

$\alpha$ in Eq. (15) is obtained as follows when COR = 0 for a broken or smashed hailstone during impact:

17 $$\alpha ={(1-COR)}^{2}$$

Although in the present experimental study the dent diameter is visible after the collision on steel plates, it is challenging to gauge the exact value of the dent diameter (${D}_{d}$) manually without a digital 3D scanner. The compressive area determines the coefficient $\frac{2{R}_{c}}{\varphi }$. As shown in Table 1, the β coefficient is derived from the distribution of the steel plate thickness and the hailstone diameter. A steel plate's compressive radius is defined as ${R}_{c}$, while its elliptical dent radius is r.

**Table 1:** β coefficients based on hail diameter and board thickness.

Steel sheet thickness (mm)	Hailstone diameters (mm)		
	38	45	50
0.3	0.90	0.90	0.99
0.45	0.83	0.77	0.83
0.6	0.85	0.78	0.90
0.7	0.81	0.75	0.80
0.8	0.80	0.77	0.85
1.0	0.69	0.74	0.81

Knud Thomsen's formula is used to approximate the surface of an ellipsoid based on the lengths of the semiaxes in Eq. (7) to correct the plastic energy. With Knud Thomsen's formula, the flat area ($${A}_{0}=\pi (2RD-{D}^{2})$$) remains the same before impact, and the deformed area ($${A}_{f})$$ resembles that of a flattened spheroid described as follows:

18 $${A}_{f}=4\pi {\left(\frac{(a{b)}^{p}+(a{c)}^{p}+(b{c)}^{p}}{3}\right)}^\frac{1}{p}$$

where $p=1.6075$, $a=b=r$ and $c=D$. Assuming a half-ellipsoid, the elliptical dented radius (r) is computed as $r=\sqrt{{R}^{2}-{(R-D)}^{2}}$, where R and D are the radius of the artificial hailstone and the dent depth, respectively. Thus, Eq. (18) can be rewritten as follows:

19 $${A}_{f}=2\pi {\left(\frac{{r}^{3.215}+2{(rD)}^{1.6075}}{3}\right)}^{1/1.6075}$$

The revised area after the collision is

20 $${\Delta A}_{r}={A}_{f}-{A}_{0}$$

21 $$\Delta {A}_{r}=2\pi {\left(\frac{{r}^{3.215}+2{(rD)}^{1.6075}}{3}\right)}^{1/1.6075}-\pi {r}^{2}$$

Hence, ϰ $= \frac{{\Delta A}_{r}}{{\Delta A}_{i}}$ and ${\Delta A}_{i}=\pi {D}^{2}$. The revised plastic energy equation is as follows:

22 $${E}_{P}=\varkappa \pi {D}^{2}{\sigma }_{Y}t$$

Dent depths of the specimens in Table 3 were predicted using the equation proposed by Uz et al.^23^ as follows:

23 $$D=\sqrt{\frac{\alpha }{\varkappa }\times \frac{m{V}^{2}}{2t\pi {\sigma }_{y}}-\frac{\beta }{\varkappa }\times \frac{{\sigma }_{y}\pi {\varphi }^{4}{l}^{3}}{32E{t}^{2}{h}^{3}}}$$

The yield stress ${(\sigma }_{Y})$, Young's modulus (E), average effective length (l), and transversal length (h) are kept constant under laboratory conditions (${\sigma }_{Y}=320\mathrm{ MPa}$, E = 200 GPa, l = 148.7 mm, h = 1 m). The coefficient ϰ is dependent on the ratio between the radius of the artificial hailstone and the elliptical radius of the damaged area. Accordingly, as the ratio of the radiuses increases, so does the coefficient determined by the ratio of ${\mathrm{\Delta A}}_{\mathrm{r}}$ and ${\mathrm{\Delta A}}_{\mathrm{i}}$. The ϰ coefficient is taken from the study of Uz et al.^20^ Experimental tests show that the relationship between ${R}_{c}$ and r is $r=0.58{R}_{c}$, with a tolerance of 0.2 mm. The following formula represents the relationship between the ϰ coefficient and the ratio between the radius of the artificial hailstone and the radius of the obtained indented ellipse:

24 $$\varkappa =1.3923{\left(\frac{R}{r}\right)}^{0.6304}$$

For a particular impact energy, Johnson and Schaffnit^24^ suggested that the dent depth is inversely related to the square of the panel thickness. In Eq. (23), the dent depth can be calculated by combining the plastic energy and the rebounding energy as well as the vibration energy and the compressive area.

### Hail impact model validation

**Figure 4:**
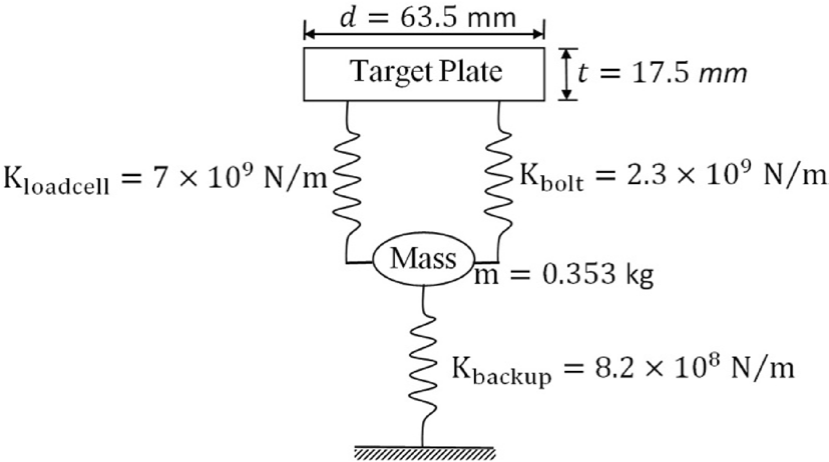
Schematic representation of the steel cylinder (load cell) and supporting springs.

The experimental test results obtained by Carney et al.^25^, which investigated the behavior of ice during impact, are used as a new validation of the FE models in this study. Cylindrical ice projectiles with diameters of 17.5 mm and lengths of 42.2 mm were used for impact tests applied on a rigid plate. To measure the force over time for each test, a force transducer device was placed behind the rigid plate. To obtain consistent data from the experimental test, the plate is set up with a similar geometry and rigidity using a 3D analytic rigid shell. With the help of three discrete spring elements, as shown in Fig. 4, the target plate of each test condition is fully modeled based on the modal characteristics. The spring elements were described by following the line of action axis in the FE models. In the boundary condition of the FE models presented in this study, the target plate is unrestrained to move only through the spring elements during impact.

**Figure 5:**
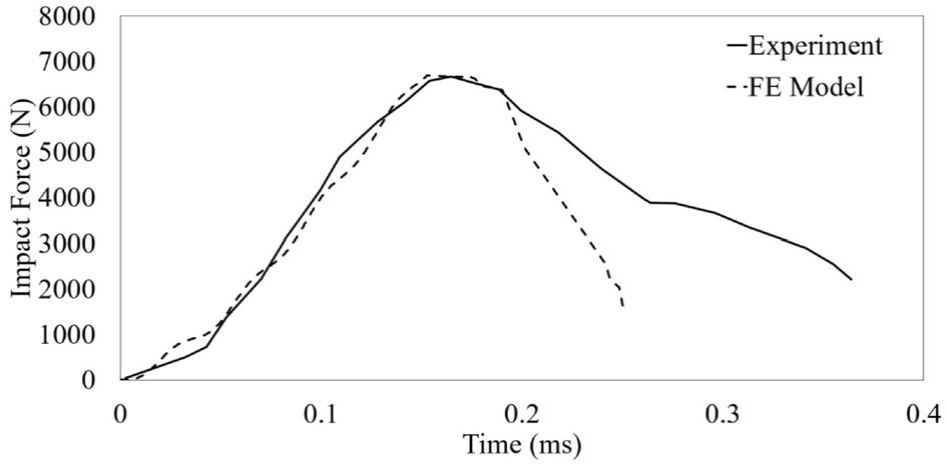
Time histories of impact force obtained by this study in comparison with the one performed by Carney et al.^25^.

**Figure 6:**
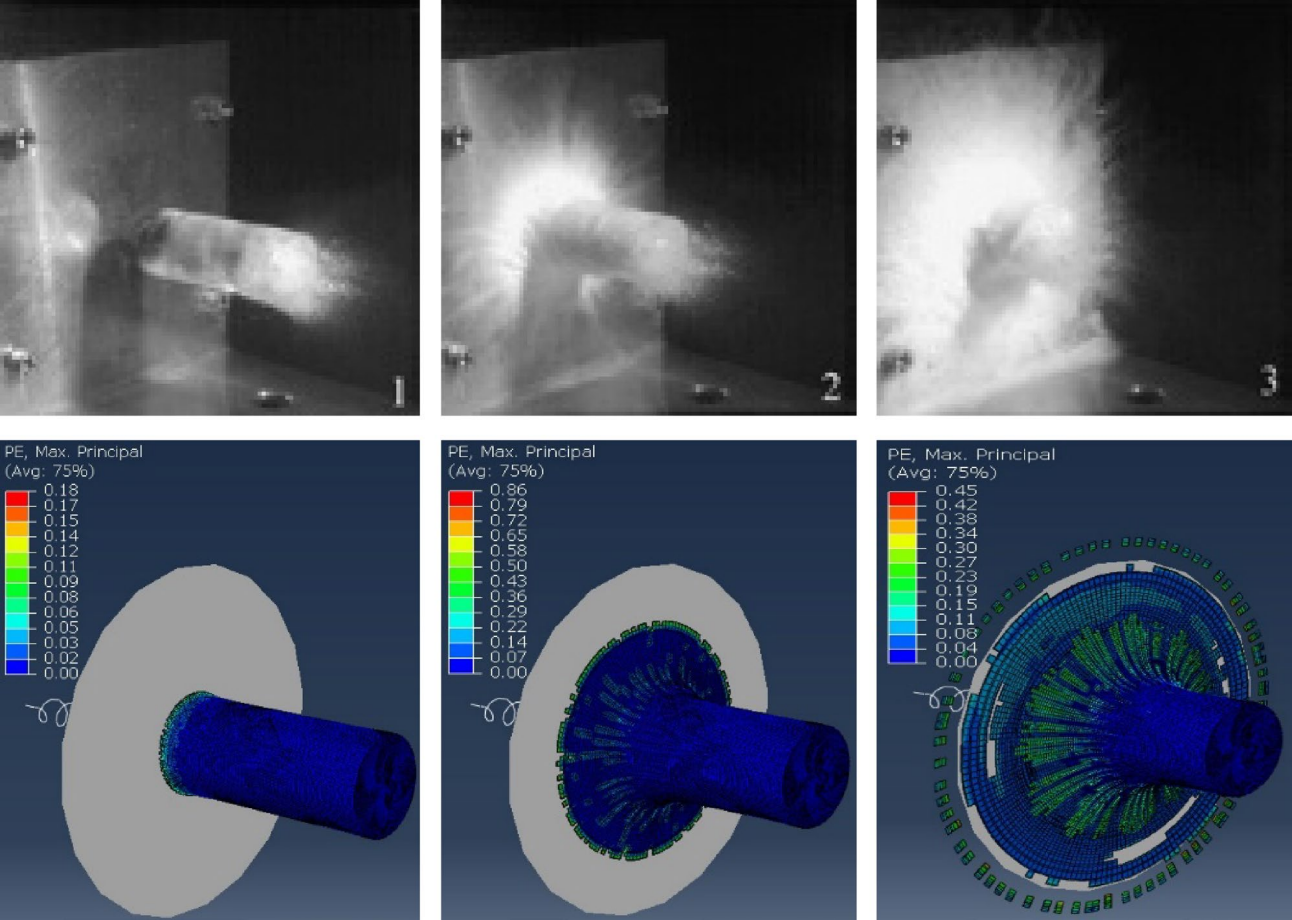
Comparison of the impact captured by experimental results obtained by Carney et al.^25^ and FE models.

The impact forces recorded over time from the force transducer device are matched with those extracted from FE models. As shown in Fig. 5, compared to the impact test from Carney et al.^25^ with 62.5 mm diameter hail at 152.4 m/s, the FE model captures the system well based on the trend and maximum impact force. The test setup for capturing the impulse in the test of Carney et al.^25^ is compared with the FE model given in Fig. 6. The material property values for hailstones are 9.38 GPa for Young's modulus, 0.33 for Poisson's ratio, 5.2 MPa for yield stress and 0.517 MPa for hydrostatic cutoff stress. The compressive strength of artificial hailstones does not noticeably affect the prediction of the maximum dent depth of steel plates by assuming that the energy conservation (vibration and plastic energy) of hailstones is negligible^21^.

The tabular representation of the strain sensitivity of the hailstone used in the current study corresponds to the data in the study by Uz et al.^21^ The strain rate determines the failure of compression.

## Results

Table 2 shows the material properties of steel plates of different thicknesses impacted by PVA and nitrogen hail balls. The velocities $\left({\mathrm{V}}_{\mathrm{c}}\right)$ measured by cameras are matched with the velocities $\left({\mathrm{V}}_{\mathrm{s}}\right)$ measured by sensors. Due to the difference between the determined camera speed and the sensor speed, the final speed is compared with the average speed. In each test, the density determined by the proposed methods agrees well with that of the natural hailstone, which remained intact after impact. Table 3 shows the measured dent depth from the experimental test, the proposed dent depth from Eq. (23) and the dent depth obtained by the FE model in the current study for each specimen, including the dent diameter on the sheet after impact. A mean professional factor of 1.09 and a coefficient of variation (COV) of 0.132 are obtained from the ratio of dent depth calculated by Eq. (23) and FE model. This study shows that the theory gives more accurate results than the FE model. Indentation depths for the three specimens are underestimated by up to 14% (i.e., 1/0.88 for specimen 889). Furthermore, the theory overestimates the indentation depth by a thickness of 0.6 mm. In specimens 1052 and 1055 made by nitrogen, the theory overestimated the dent depth due to major fragmentation of hailstones after impact. However, specimen 910, which did not break after impact, was made by PVA, although the theory for that specimen is also overestimated. The reason for this is thought to be because of the lower density for the related specimen. For each specimen, the dent diameters (Dx and Dy) in the longitudinal and transverse directions are measured after impact to determine the β coefficients given in Table 1. A mean professional factor of 0.99 and a coefficient of variation (COV) of 0.114 are obtained by using the FE model. Based on the measured dent depth on the tested sheet, it appears that the FE model provides an underestimation.

**Table 2:** Measured data properties of hailstones used in this study before impact tests.

Properties	Specimen no	Plate thickness (mm)	Hailstone diameter (mm)	Mass (g)	Density (kg/m^3^)	Sensor velocity (m/s)	Camera velocity (m/s)	Average velocity (m/s)	Kinetic energy (J)
Nitrogen	1050	0.6	44.94	51.2	1077.6	23.32	27.41	25.36	16.47
Nitrogen	1052	0.6	48.99	71.6	1163.0	23.37	28.29	25.83	23.89
Nitrogen	1053	0.6	49.25	61.9	989.8	22.52	25.47	23.99	17.82
Nitrogen	1054	0.6	48.02	58.7	1012.2	27.72	28.48	28.10	23.18
Nitrogen	1055	0.6	46.37	56.8	1088.0	24.81	29.17	26.99	20.69
PVA	931	1	50.00	64.6	953.4	27.92	30.06	28.99	27.15
PVA	937	1	50.00	63.9	975.3	30.87	36.74	33.80	36.51
PVA	940	0.8	50.00	64.1	964.6	21.46	33.06	27.26	23.82
PVA	889	0.8	45.00	44.5	1016.8	32.80	28.69	30.75	21.04
PVA	896	0.8	45.00	49.3	993.4	25.86	27.33	26.59	17.43
PVA	897	0.8	45.00	45.9	982.4	24.92	27.55	26.24	15.80
PVA	882	0.7	45.00	47.4	1005.9	25.75	28.69	27.22	17.56
PVA	883	0.7	45.00	44.8	1005.8	25.95	31.56	28.75	18.52
PVA	885	0.7	45.00	48.9	1045.0	28.80	30.06	29.43	21.17
PVA	871	0.6	45.00	46.5	1081.9	29.16	33.40	31.28	22.74
PVA	910	0.45	45.00	45	973.5	28.88	30.06	29.47	19.54
PVA	912	0.45	45.00	46.4	961.3	26.05	28.69	27.37	17.38
PVA	927	0.3	45.00	44.5	941.4	22.42	28.69	25.56	14.53
PVA	1011	0.6	38.00	27.4	1001.1	21.87	27.33	24.60	8.29
PVA	1012	0.6	38.00	27.5	993.6	28.07	28.90	28.49	11.16
PVA	1015	0.6	38.00	28.3	1029.3	29.12	30.06	29.59	12.39

**Table 3:** Measured dent depths and diameters on the steel sheets after impact.

Specimen no	Rebounded velocity (m/s)	Dx (mm)	Dy (mm)	Average dent diameter (mm)	Proposed dent depth (mm)	FEM (5.17 kPa)	Depth, Dz (mm) Gauge	Rate	Projectile integrity
1050	0.53	39.39	42.05	40.7	2.73	3.03	2.80	0.90	Whole
1052	0.91	36.84	35.92	36.4	3.02	2.22	2.67	1.36	Major
1053	1.47	36.45	33.65	35.1	2.60	2.60	2.23	1.00	Minor
1054	0.02	38.39	40.48	39.4	3.01	2.99	2.82	1.01	Whole
1055	0.01	44.38	44.39	44.4	2.94	2.18	2.67	1.35	Major
931	0.26	38.94	49.58	44.3	2.79	2.70	2.68	1.03	Minor
937	0.78	52.57	60.49	56.5	3.28	3.37	3.28	0.97	Whole
940	1.38	44.49	43.73	44.1	3.13	2.93	3.51	1.07	Whole
889	1.38	31.99	31.24	31.6	2.83	2.56	2.34	1.11	Minor
896	1.01	35.07	33.20	34.1	2.59	2.94	2.51	0.88	Whole
897	1.12	33.48	34.60	34.0	2.46	2.32	2.31	1.06	Whole
882	1.07	33.87	35.34	34.6	2.66	2.60	2.40	1.02	Whole
883	1.22	36.48	36.51	36.5	2.81	3.08	2.74	0.91	Whole
885	1.00	33.65	31.81	32.7	3.03	3.03	2.78	1.00	Whole
871	0.89	36.66	38.48	37.6	3.48	3.15	3.18	1.10	Whole
910	1.35	33.07	35.23	34.2	3.25	2.58	2.95	1.26	Whole
912	1.21	33.48	33.45	33.5	2.99	3.02	2.94	0.99	Whole
927	1.61	43.75	45.58	44.7	3.74	3.60	3.87	1.04	Whole
1011	1.10	30.43	28.03	29.2	2.11	1.95	2.13	1.08	Whole
1012	1.25	33.67	36.58	35.1	2.40	2.06	2.31	1.17	Whole
1015	0.81	38.09	37.12	37.6	2.55	2.19	2.40	1.16	Whole
Mean								1.09	
COV								0.132	

**Figure 7:**
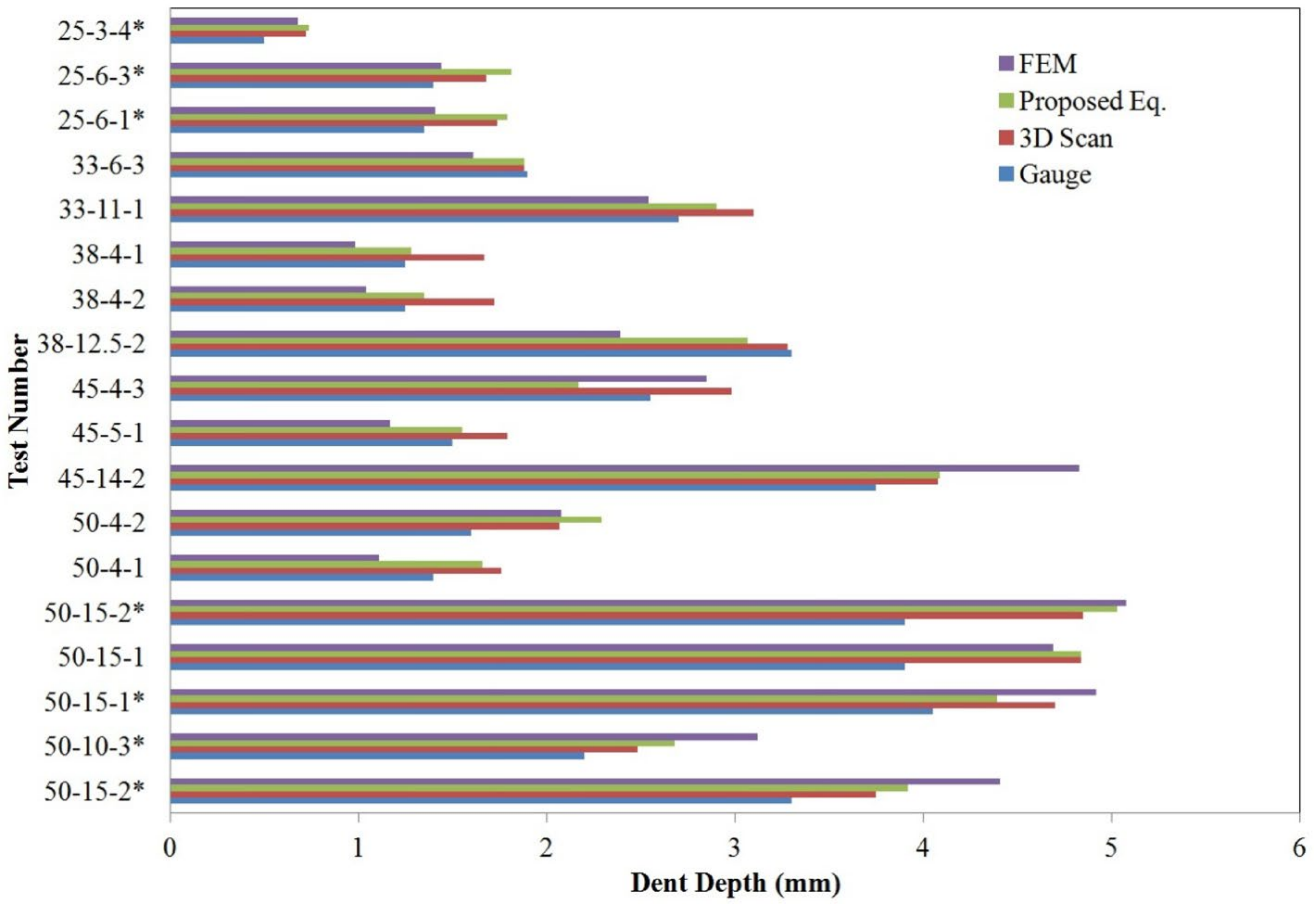
Comparison between the FE model and Wu’s^8^ results.

**Figure 8:**
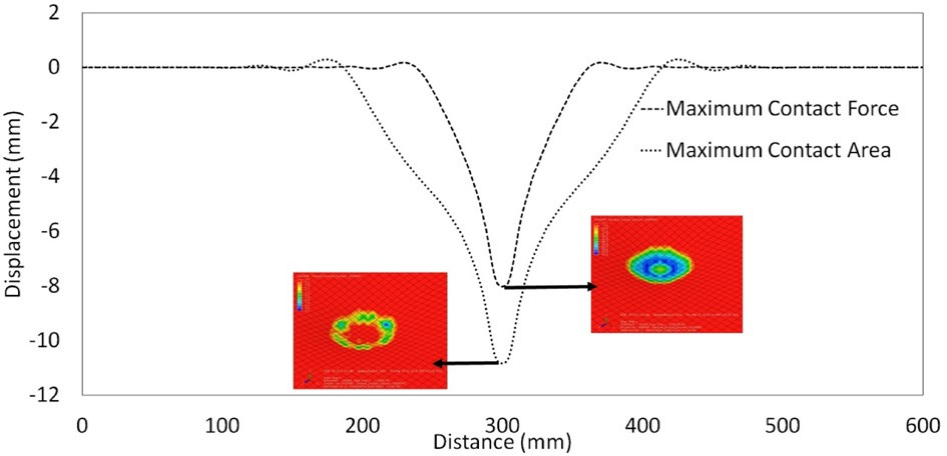
Displacement results of the FE model for specimen 50-15-1.

Equation (23) gives a mean professional factor of 1.06 and a coefficient of variation (COV) of 0.055. Some of the tests conducted by Wu^8^ are performed in the FE model of this study. Figure 7 shows a comparison between the dent depths determined with the FE models, with the gauges and 3D scans in the study of Wu^8^ and with the equation proposed by Uz et al.^21^ Except for the samples with a hailstone diameter of 25 mm, the results of the FE models are not even in the range of the results measured with gauges and 3D scans. Due to the limited scope of this article, there is only one FE model result presented in Fig. 8, which refers to sample 50-15-1. A hailstone with a diameter of 50.2 mm is launched at a speed of 35.6 m/s into a steel sheet of 0.55 mm thickness.

**Figure 9:**
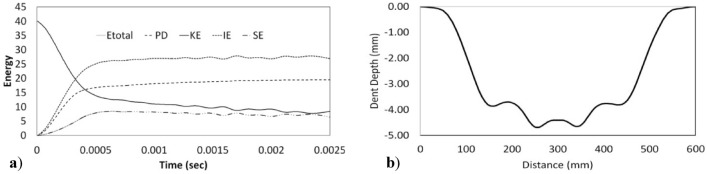
Results of the FE model for specimen 50-15-1: (**a**) energy history and (**b**) permanent distortion (springback modeling).

**Figure 10:**
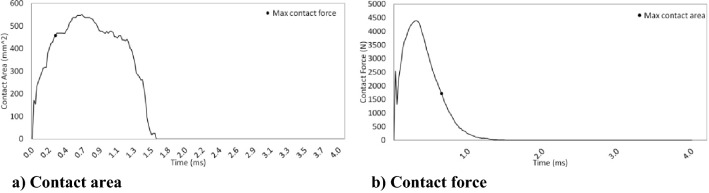
Time histories of the impact of the hailstone on specimen 50-15-1.

The bend of the sheet in the middle of the impact is shown in Fig. 8. As the impact force increases, the deflection of the steel increases until the highest contact is reached between the steel sheets and the hailstones. The dent depth of 4.69 mm determined in the FE model agrees with the dent depth of 4.84 mm determined by both the experimental test performed by Wu^8^ and the equation proposed by Uz et al.^23^. Figure 9b shows the deformation resulting from the process of the 0.55 mm thick steel sheet in the springback model connected to the successfully finished impact model in Abaqus software. Although the hailstone reached the upper limit force at 0.37 ms (Fig. 10b), it continued to travel in the vertical direction at 0.91 ms. The hailstone and steel sheet were in close contact at 1.5 ms (Fig. 10a). Figure 9a shows the energy dissipation over the course of the impact. Figure 9a shows that the energy dissipated by rate-independent plastic deformation (PD) remained at approximately 15 J, while the kinetic energy remained at approximately 10 J due to the rebound of the hailstone after impact.

The total strain energy (IE) and recoverable strain energy (SE) show the accuracy of the theoretical dent depth equation of this current study.

During the impact, a hailstone with a diameter of 50.2 mm deformed and formed a contact area of 552 mm^2^.

Equation (23) is independent of time for the vibration energy calculation. The stress‒strain graph of the material shows the end of linear performance followed by a linear trend until springback begins. Based on the toughness of the material, a time-independent equation is used to calculate the plastic energy. According to the current literature, there is a linear relationship between the impact energy and indentation depth, while the indentation depth and material thickness have an inverse relationship.

### Resistance factor

Because of common practices in the field, researchers have included this section despite their reservations about the reliability analysis procedure. The reason is that it is commonly used in the literature. This section includes the determination of the resistance factor that can be abused. The reliability analysis methodology and statistical parameters used in this paper were adopted from Driver et al.^26^ and Teh and Uz^27^, who determined the required resistance factor ${\varnothing }_{r}$ using the equation proposed by Fisher et al.^28^.

25 $${\varnothing }_{r}=\left(0.0062{\beta }_{r}^{2}-0.131{\beta }_{r}+1.338\right){M}_{m}{F}_{m}{P}_{m}{e}^{-p}$$

26 $$p={\alpha }_{r}{\beta }_{r}\sqrt{{V}_{m}^{2}+{V}_{F}^{2}+{V}_{P}^{2}}$$

In Eq. (25), $${M}_{m}$$ and $${F}_{m}$$ are the mean values of the material factor and the manufacturing factor, which are 1.11 and 1.00. In the present work, the mean value of the professional factor ($${P}_{m})$$ is given in Table 3. In Eq. (26), the separation variable $${\alpha }_{r}$$ is equal to 0.55, while $${V}_{m}$$, $${V}_{F}$$ and $${V}_{P}$$ are the coefficients of variation based on the material, manufacturing factors and the current professional factor given in Table 3, respectively, which are assumed to be 0.054, 0.05 and 0.132.It was found that to achieve the target reliability index $${\beta }_{r}$$ of 4, a resistance factor $${\varnothing }_{r}$$ of 0.80 is required for Eq. (23).

## Conclusions

The following factors are considered in this study: sheet thickness, material thickness, batten spacing, distance of the dent center of gravity from the closest edge and hail size. For inelastic steel, the dent depth is independent of the hailstone diameter. A smaller hailstone with a larger impact produces a similar permanent dent and the same impact energy as a larger hailstone with a smaller impact. The plastic distortion noted with the proposed equation agrees well with the experimental results. The dent depth in this case is not inversely proportional to the square root of the plate thickness or to the vibration-induced yield stress. The FE models were not able to provide accurate predictions of deformation depths shown in experiments compared to analytical models. Numerical models were able to accurately represent approximately 82% of the experimental results of permanent deformation of the plate. The elastic response of steel plates is generally overestimated by FE models. It is also necessary to conduct a separate study to accurately determine the strain sensitivity of the artificial hailstone in relation to the hardness of the hailstone, which determines the impact force at the point of contact. Based on these findings, new design methods for hail-resistant steel sheets can be developed that have a wider range of applications. A resistance factor of 0.80 is recommended for use with the equation in order to achieve the target reliability index of 4. Relative to the current AISC specification’s equation, which has a resistance factor of 0.75 as specified in the code, the use of the proposed equation will facilitate structural design that are more economical yet reliable.

## Data Availability

The datasets generated and/or analyzed during the current study are available on request from the corresponding author.
